# NMDA Receptors: Distribution, Role, and Insights into Neuropsychiatric Disorders

**DOI:** 10.3390/ph17101265

**Published:** 2024-09-25

**Authors:** Marie Beaurain, Anne-Sophie Salabert, Pierre Payoux, Emmanuel Gras, Franck Talmont

**Affiliations:** 1 ToNIC, Toulouse NeuroImaging Center, INSERM, UPS, Université de Toulouse, 31024 Toulouse, France; beaurain.m@chu-toulouse.fr (M.B.); anne-sophie.salabert@inserm.fr (A.-S.S.); pierre.payoux@inserm.fr (P.P.); 2 Laboratoire Hétérochimie Fondamentale et Appliquée (LHFA, UMR 5069), CNRS, UPS, Université de Toulouse, 118 Route de Narbonne, CEDEX 9, 31062 Toulouse, France; emmanuel.gras@univ-tlse3.fr; 3 Institut de Pharmacologie et de Biologie Structurale (IPBS), CNRS, UPS, Université de Toulouse, 31077 Toulouse, France

**Keywords:** NMDA receptors, neuropsychiatric disorders, NMDA channel blockers

## Abstract

Background: N-methyl-D-aspartate receptors (NMDARs) are members of the ionotropic glutamate receptor family. These ligand-gated channels are entwined with numerous fundamental neurological functions within the central nervous system (CNS), and numerous neuropsychiatric disorders may arise from their malfunction. Methods: The purpose of the present review is to provide a detailed description of NMDARs by addressing their molecular structures, activation mechanisms, and physiological roles in the mammalian brain. In the second part, their role in various neuropsychiatric disorders including stroke, epilepsy, anti-NMDA encephalitis, Alzheimer’s and Huntington’s diseases, schizophrenia, depression, neuropathic pain, opioid-induced tolerance, and hyperalgesia will be covered. Results: Finally, through a careful exploration of the main non-competitive NMDARs antagonists (channel-blockers). Conclusion: We discuss the strengths and limitations of the various molecular structures developed for diagnostic or therapeutic purposes.

## 1. Introduction

Glutamate receptors (GluRs) are ubiquitously distributed throughout the central nervous system (CNS). They are involved in a diverse range of neurological functions, including but not limited to pain transmission, neuronal growth, motor function, long-term memory processes, and synaptic plasticity [1]. They are activated by glutamate, the amino acid acting as the main excitatory neurotransmitter found in the body. These receptors are classified into two distinct groups, namely metabotropic receptors (mGluRs) and ionotropic receptors (iGluRs).

Metabotropic glutamate receptors are G-protein-coupled receptors (GPCR). They are classified into three distinct groups based on sequence homology, signal transduction, and pharmacological profiles. According to [2], group I comprises mGluR1 and mGluR5, while group II comprises mGluR2 and mGluR3, and group III comprises mGluR4, mGluR6, mGluR7, and mGluR8. The diverse roles played by mGluRs are reflected in the diverse locations they are found in, including but not limited to the glutamatergic synapse and glial cells, including astrocytes, oligodendrocytes, and microglia. The main characteristic of these receptors is their slow activation rate, which suggests that they respond to prolonged exposure to glutamate. They are therefore responsible for slowing down synaptic responses and can lead to the integration of time-dispersed signals. At the same synapse, glutamate can trigger fast synaptic responses (via iGluRs) and regulate or adjust neuronal activity (via mGluRs) [3,4].

iGluRs are ligand-gated ion channels and are divided into three subtypes based on their pharmacological properties: NMDA (N-methyl-D-aspartate receptors) receptors (NMDARs), AMPA (α-amino-3-hydroxy-5-methylisoxazole-4-proprionic acid) receptors (AMPARs), and kainate receptors (KARs). They are composed of four subunits, assembled into homomers or heterotetramers, which form a central channel permeable to cations (Na^+^, K^+^, and Ca^2+^) [5].

In contrast to mGluRs, iGluRs are accountable for rapid excitatory synaptic transmission. Their fast kinetics allow them to respond to a brief release of glutamate in the synaptic cleft. Among the three principal classes of iGluRs, AMPARs and NMDARs are predominantly post-synaptic, whereas KARs are located pre- or extra-synaptically and appear to play a modulatory role in synaptic transmission that is comparable to that of mGluRs. Although all iGluRs can be activated by short exposure to glutamate, they have distinct kinetic properties. Within an excitatory synapse, two components of the excitatory post-synaptic current can be distinguished: a fast component mediated by AMPARs and a slow component promoted by NMDARs [6]. As well as other iGluRs, NMDARs are heteromeric multimers composed of three types of subunits: GluN1, expressed as eight splice variants, GluN2, and GluN3, encoded by four (GluN2A-D) and two (GluN3A-B) genes, respectively. Functional NMDARs are required to comprise two GluN1 subunits and two GluN2 and/or GluN3 subunits.

Each subunit has a similar architecture, described as follows:

- An extracellular amino-terminal domain (ATD) containing the ligand agonist binding domain (LBD or ABD) as well as allosteric modulation sites.
- The transmembrane domain (TMD) that comprises three segments, namely M1, M3, and M4, and a reentrant intramembrane loop that faces the cytoplasm (M2), thereby forming the ion channel and selectivity filter.
- An intracellular C-terminal domain (CTD) that varies in size depending on the subunit and influences membrane targeting and couples to intracellular signaling molecules.

**Figure 1:**
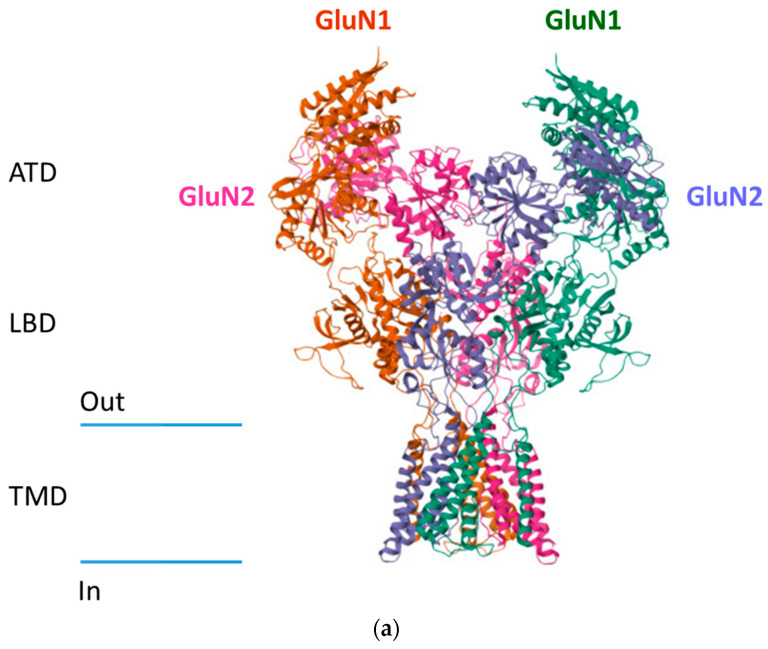

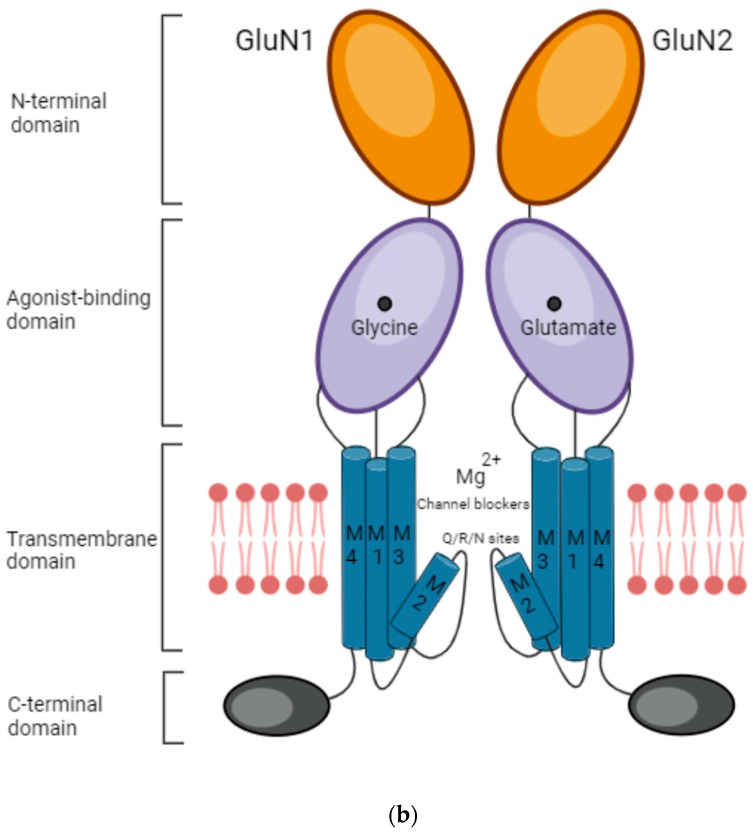
Structure of NMDA receptors. (**a**) Cryo-EM structure of GluN1/GluN2B NMDA receptor in the glutamate/glycine-bound conformation (pdb 5IOU) https://doi.org/10.1016/j.cell.2016.03.028. (**b**) Schematic representation of NMDA receptors.

The binding of glutamate or an agonist to the LBD leads to a conformational change in the TMD of the receptor, leading to the opening of the ion channel that triggers cation influx [7] (Figure 1a,b).

A key determinant of the functioning of iGluRs is the highly conserved Q/R/N (glutamine/arginine/asparagine) site. This site, which controls Ca^2+^ permeability and blocking by magnesium ions (Mg^2+^) [8], is often referred to as the selectivity filter. This site is located on the top of the M2 re-entrant loop. The ion channel is divided into two parts: an intracellular domain, lined by the M2 re-entrant loop, and an extracellular domain, formed by the M3 transmembrane helix. The extracellular domain is separated by a narrow constriction, located on the top of the M2 re-entrant loop (i.e., at the Q/R/N site) [9]. This region is significantly associated with the ionotropic properties of the receptor. Hence, an amino acid substitution at the Q/R/N site of GluN2 will have minimal impact on Ca^2+^ permeability but significantly reduces the obstruction of the channel by Mg^2+^ ions. Conversely, a substitution of the asparagine residue of GluN1 reduces Ca^2+^ permeability without affecting Mg^2+^ blockage [10,11]. The various composition in receptor subunits may have an impact on numerous parameters, including the kinetics of receptor inactivation, receptor opening and permeability to cations, sensitivity to agonists, and blocking of the channel by the Mg^2+^ ion [12].

**Figure 2:**
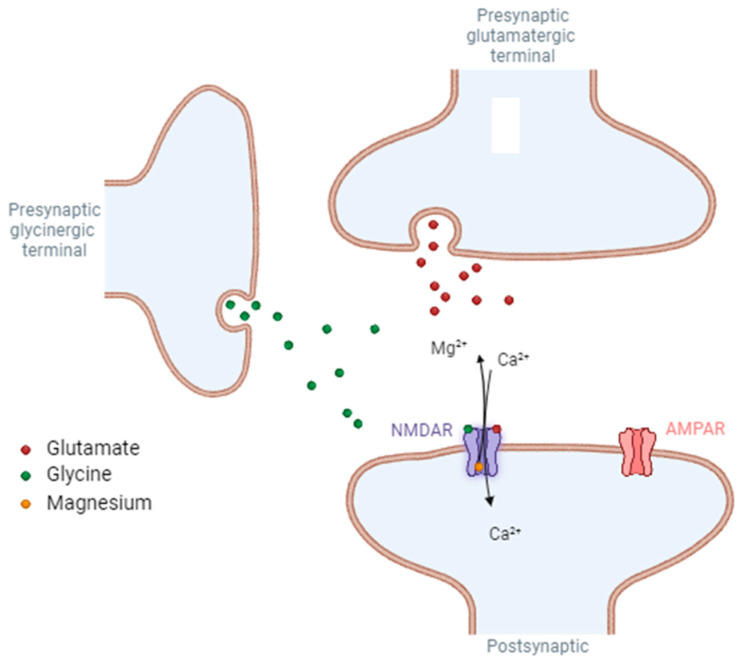
Schematic representation of NMDA receptor activation. NMDAR, N-methy-D-aspartate receptor; AMPAR, α-amino-3-hydroxy-5-methyl-4-isoxazolepropionic acid receptor.

In contrast to the majority of other neurotransmitter receptors, NMDARs, comprised of the GluN1 and GluN2 subunits, necessitate the activation by two agonists (Figure 2).

The first, glutamate, binds to the GluN2 subunit. The second, also known as the co-agonist, can be either glycine or D-serine and binds to the GluN1 subunit. Papouin et al. demonstrated the importance of this co-agonist. Indeed, D-serine acts as a co-agonist for synaptic receptors, while glycine is an extrasynaptic receptor co-agonist [13]. Astroglial cells in contact with excitatory synapses produce and release D-serine. These non-neuronal cells therefore tightly control the activity of synaptic NMDARs and the resulting phenomena [14,15]. The NMDAR composed of GluN1/GluN3 subunits only requires glycine to be activated [12] and therefore is not considered to be a glutamate receptor [16]. Once glutamate and the co-agonist have been bound to their respective binding sites, activation of the receptor also requires membrane depolarization. This depolarization removes the blockage exerted by magnesium ions (Mg^2+^) from the channel. At resting membrane potential (around −70 mV), extracellular Mg^2+^ ions block the channel associated with the receptor. As the dissociation constant of magnesium is an exponential function of membrane potential, subsequent to neuronal depolarization, the negative electrostatic forces that attracted the Mg^2+^ ion to the GluN2 subunit collapse, resulting in the release of the cation [17]. Due to the glutamate-dependent activation of Ca^2+^-impermeable AMPAR, membrane depolarization occurs, thus allowing the influx of Na^+^ ions. The depolarization triggers the removal of Mg^2+^ from the NMDAR pore to facilitate its activation. Therefore, activation of AMPA receptors by glutamate precedes activation of NMDA receptors [18]. This dual dependence on distinct and independent activation mechanisms explicates the delayed activation of NMDARs in contrast to AMPA channels, for instance. The slow activation is a reflection of the prolonged residence time of glutamate on NMDARs, which serves as a detector for the congruity of the excitation of the pre- and postsynaptic elements. These constituents pertain to biological membranes, and subsequent to its release from the presynaptic component, glutamate binds to the receptor. However, the blocking by Mg^2+^ prevents any ionic flow at the resting electrical potential. Thus, if other glutamatergic afferents excite the post-synaptic neuron, the resulting depolarization can transiently remove the Mg^2+^ block. If this occurs while glutamate is still bound to NMDARs, then cations will pass through the ion channel. The cationic permeability of NMDARs then causes an influx of Na^+^, K^+^, and, most importantly, Ca^2+^ ions, which allows the initiation of neuronal plasticity, which is essential for learning and memory. Unlike other glutamatergic receptors, NMDARs exhibit slow activation and inactivation kinetics. Since this activation pattern involves the slow dissociation of glutamate, NMDARs have a higher affinity for glutamate than other iGluRs (EC50~1 μM, almost one hundred times higher than AMPARs, EC50~100 μM) [3,6,19]. Aside from receptor activation phases, the reuptake system (excitatory amino acid transporters) located in the presynaptic element and glial cells maintains the concentration of glutamate at nanomolar levels. After vesicular release, the concentration of glutamate in the synaptic cleft can peak at around one mM before rapidly falling (one ms) due to diffusion and reuptake. This brief increase in concentration allows a temporary opening of NMDARs, which is necessary for physiological synaptic transmission [12]. The extracellular concentration of Na^+^ ions varies from 145 to 150 mM, while the concentration of Ca^2+^ ions is between 1 and 1.5 mM. The relative permeability of the NMDAR ion channel for Ca^2+^ ions is ten times greater than that of Na^+^ ions, resulting in a significant inflow of Ca^2+^ ions into the cells upon receptor opening. These Ca^2+^ ions are a crucial component of intracellular signaling which is implicated in numerous forms of neuronal plasticity, and they also act as mediators of excitotoxic cell death when present in excess [12,20]. The activation of NMDARs goes along with one of the other glutamatergic receptors, such as AMPARs or metabotropic receptors. Indeed, the increase in intraneuronal calcium concentration induced by channel aperture is amplified by the opening of voltage-gated Ca^2+^ channels and the release of inositol triphosphate (IP3) promoted from metabotropic receptor activation. This calcium entry is the starting point for the synthesis of second and third messengers such as prostaglandins and nitric oxide (NO), which in turn facilitate the presynaptic release of glutamate [21].

The aim of this review is to highlight the structural complexity of the NMDAR in the context of glutamate receptors in relation to the complexity of their physiological and pathological role. We show that this apparent complexity has ultimately resulted in a limited number of drugs and that they almost all target a restricted structural region of the receptors.

## 2. Role of NMDA Receptors

NMDARs play a crucial role in the mammalian brain. They underlie synaptic plasticity phenomena such as long-term potentiation (LTP) and long-term depression (LTD), which are the cellular mechanisms of memory and learning [22]. They are also involved in the phenomenon of excitotoxicity, which is responsible for the neuronal death observed in several neurodegenerative diseases such as Alzheimer’s disease and amyotrophic lateral sclerosis [23]. In adult brains, the distribution of NMDARs exhibits heterogeneity both within brain regions and on the surface of neurons. Although predominantly postsynaptic, NMDARs can also be found at extrasynaptic sites, where they can be recruited to substitute synaptic receptors [1]. NMDARs can also be found on the presynaptic membrane, where neurotransmitter release can be modulated [24], and on glial cells, particularly oligodendrocytes [9]. Extensive studies have been conducted in rodents on the expression profiles of different subunits during development [25]. Subunit composition varies according to receptor location, although there is no systematic distribution of subunits according to subcellular region, synaptic vs. extrasynaptic [26]. The GluN1 subunit, which is present in all functional NMDARs, is ubiquitous throughout the CNS, from embryonic to adult stages. NMDAR splicing variants are detected, depending on the stage of development and location in the CNS, but the functional consequences of differential expression of GluN1 isoforms are still poorly understood [25]. The four GluN2 subunits, which are the primary contributors to the functional heterogeneity of NMDARs, exhibit a distinct temporal expression profile. The embryonic brain contains only GluN2B and D subunits. Expression of GluN2A appears within the initial few weeks following birth and is thereafter spread throughout all regions of the adult CNS. GluN2B expression is progressively restricted to the forebrain, while GluN2D expression is declining and remains only in the diencephalon and midbrain. The expression of GluN2C occurs later and is restricted to the cerebellum and olfactory bulb [27,28,29]. Furthermore, the expression profiles of GluN3A and B subunits are variable. GluN3A expression peaks in early postnatal life and then slowly declines, whereas GluN3B expression increases slowly throughout development into adulthood. The specific expression of GluN2B, GluN2D, and GluN3A subunits early in development suggests that these subunits are important for synaptogenesis and synaptic maturation. In the adult brain, GluN2A and GluN2B are the predominant subunits, especially in higher brain structures (such as the hippocampus and cortex), suggesting that they play a central role in synaptic function and plasticity [30,31].

## 3. Presynaptic NMDA Receptors

Although mainly found on the postsynaptic membrane, NMDARs are also found on the pre-synaptic membrane [24]. During the initial stages of neuronal development, the expression of presynaptic NMDARs (pre-NMDARs) is particularly elevated in the cerebral cortex. These receptors play an important role in the maturation of synapses and the neuronal network. However, unlike their post-synaptic counterparts, they are not ubiquitous. Instead, specific groups of neurons localize pre-NMDARs, and they display activation specificities, suggesting that they contribute distinctly to regulating synaptic activity and information processing. In rodents, the cerebral cortex, hippocampus, amygdala, striatum, nucleus accumbens, cerebellum, brainstem, spinal cord, and peripheral nervous system contain pre-NMDARs [32]. As there is no absolute rule for the subunit composition of presynaptic NMDA receptors, it is likely that they will contain subunits that are highly expressed at this early stage, such as the GluN2B or GluN3A subunits [32]. Depending on the source of the glutamate that activates them, pre-NMDARs can act as autoreceptors or heteroreceptors. When activated by glutamate released from the presynaptic element, they are referred to as autoreceptors. Contrarily, they are referred to as heteroreceptors when the activating glutamate originates from nearby synaptic terminals or astrocytes. They can also be activated by the retrograde release of glutamate, although this mode of activation has only been demonstrated in the cerebellum [33]. The activation of pre-NMDARs results in pre- or postsynaptic effects through distinct signaling pathways. In most synapses, the activation of pre-NMDARs results in a Ca^2+^ entry, thus stimulating the fusion of vesicles within the membrane. This phenomenon has been demonstrated with the observation that synapses between cerebellar interneurons and Purkinje cells initiate the release of GABA, thereby enhancing the frequency of inhibitory postsynaptic currents [34]. Furthermore, pre-NMDARs activation can also have direct effects on axon excitability or act on the postsynaptic element via diffusion of NO across the synapse (anterograde signaling) [32].

## 4. Extrasynaptic NMDA Receptors

The NMDARs located more than 100 nm from the postsynaptic density are considered to be extrasynaptic [35]. In adults, these receptors are distributed along dendrites as well as on dendritic spines and are in close contact with adjacent structures, including glia, axons, and their terminals. They are in contact with ambient glutamate of glial origin, the concentration of which is maintained at low levels by glutamate transporters. This concentration is not sufficient to activate NMDARs, but it may explain the lifespan of weak tonic currents [36,37]. Their activation can be caused by excess glutamate released from neighboring synapses (especially in the case of perisynaptic NMDARs) or by ectopic glutamate release from neighboring structures such as astrocytes [38,39,40]. Numerous studies have highlighted this particular phenomenon, indicating the existence of focal points of communication between glial cells and neurons via glutamate release. This facilitates the modulation of neurotransmitter release at the axonal terminal [41,42,43,44]. These studies suggest that extrasynaptic NMDARs are organized into clusters at these communication points, resulting in a discrete and non-diffuse distribution along dendritic extensions [35]. This distribution appears to be regulated by association with scaffolding proteins such as PDZ domain proteins, GIPC, PSD-95, or SAP102, which play a stabilizing role [24]. Extrasynaptic NMDARs are mostly composed of the GluN2B and GluN2D subunits, which form heterodimers (GluN1/GluN2B and GluN1/GluN2D) or heterotrimers (GluN1/GluN2B/GluN2D). Nonetheless, the GluN2A subunit, which exhibits the highest concentration in mature synapses, has also been identified at the extrasynaptic level [45]. The properties of these receptors have been investigated in various brain structures by measuring the ion conductance and properties of the currents using electrophysiological recordings. Receptors have been found in the cerebellum, Golgi cells, and Purkinje cells [46,47,48,49,50]. They are also found in the visual cortex [51] and within the hippocampus, in the granular cells of the dentate gyrus [52,53], in the pyramidal cells of CA1 [54], and in primary cultures of hippocampal neurons [55].

Effects attributed to extrasynaptic NMDARs can be either positive, thus acting on neuronal activity, modulation, and synaptic plasticity, or negative and acting through excessive activation that promotes neuronal death. Both types of effects depend on the location of the receptors on the neuron, the receptor subtype, and the associated scaffolding and signaling proteins [56]. The beneficial effects have been demonstrated in several studies. For example, Fellin et al. showed that glutamate release from hippocampal astrocytes is responsible for the synchronous activation of CA1 neurons via the opening of extrasynaptic NMDARs, which is essential for information processing [42]. Another study by Chalifoux and Carter in 2011 on pyramidal neurons in the mouse prefrontal cortex showed that glutamate released in excess at the synapse (spillover) can diffuse outside the synaptic cleft and activate NMDARs, thereby promoting synaptic plasticity [57]. Under physiological conditions, glial and neuronal glutamate transporters [58] limit this spillover.

Furthermore, in addition to their significant role in regulating neuronal activity, there is a growing body of evidence indicating that extrasynaptic NMDARs are involved in the processes of excitotoxicity and neuronal death [35]. Excitotoxicity refers to the ability of glutamate or related excitatory amino acids to mediate the death of central neurons under certain conditions, for example, after intense exposure. John Olney proposed this concept in 1969, following earlier observations by Lucas and Newhouse in the 1950s that glutamate injections caused retinal cell degeneration [59]. John Olney later demonstrated that the lesions involved many central structures, including the hypothalamic and circumventricular brain regions [60]. Despite the fact that excitotoxicity can be mediated by multiple iGluRs, the elevated calcium conductance of NMDAR renders it a pivotal player in both acute and chronic neurotoxic processes. The uncontrolled increase in intracellular Ca^2+^ concentration resulting from excessive activation of NMDAR leads to the production of superoxide anion (O^2−^) and nitric oxide (NO), which together generate the cytotoxic reactive oxygen species peroxynitrite, a particularly oxidizing anion in oxidative stress [61]. Ca^2+^ ions can also activate cytoplasmic enzymes such as protein kinase C (PKC), phospholipases A2 and C (PLA2 and PLC), endonucleases, and proteases. They also activate the p28 MAPK protein (mitogen-activated protein kinase p38), which activates transcription factors. These phenomena, in conjunction with calcium overload in the mitochondria, facilitate the production of prostaglandin, oxidative stress, and the degradation of cellular proteins and nucleic acids. Furthermore, caspase activation links NMDARs to the physiological death of neurons (apoptosis), an essential process in brain development and synaptic plasticity but also in cell destruction in situations of aggression [62]. Several studies have demonstrated that low concentrations of glutamate (10 to 20 µM) or NMDA (10 to 20 µM) do not cause neuronal death in cell cultures [63,64]. However, the population begins to decline as the concentration increases, and maximum mortality is observed at NMDA concentrations between 50 and 100 µM [64]. Based on convergent data, it is commonly accepted that the excitotoxicity phenomenon is mainly mediated by extrasynaptic NMDARs, whereas synaptic receptors stimulate cell survival. The roles of synaptic and extrasynaptic receptors have been studied, in particular, in the gene encoding the transcription factor CREB (cyclic AMP response element binding protein), a protein involved in neuronal survival processes. In a fundamental study, Hardingham et al. [65] discovered that the stimulation of synaptic NMDARs by treatment by administering bicuculline, a GABAergic antagonist that enhances synaptic activity, induces the activation of the pro-survival protein CREB. Subsequent incubation of neurons in a bath containing a high concentration of glutamate stops CREB signaling and causes significant neuronal death [66]. Although bath incubation with glutamate is likely to activate both synaptic and extrasynaptic NMDARs, the authors suggest that activation of extrasynaptic receptors counteracts the effect of synaptic ones and suppresses the pro-survival signaling pathway. In response to this initial study, other works have attempted to isolate extrasynaptic NMDARs to investigate their role in cell death [56]. CREB phosphorylation is dependent on the activation of the Ras/ERK pathway [67]. It was demonstrated that stimulation of synaptic NMDARs activates the Ras/ERK pathway, whereas activation of extrasynaptic receptors inactivates it [68,69,70]. Nonetheless, this theory fails to encompass all the occurrences associated with excitotoxicity. Initially, extrasynaptic NMDARs dominate in young developing neurons, which are resistant to high concentrations of glutamate and NMDA [65,71,72]. Furthermore, glutamate excitotoxicity has not been observed in neurons in the rat retinal ganglion cell layer, where synaptic NMDARs are not expressed and where current is mediated only by extrasynaptic receptors [65,71,72,73,74]. This statement has also been subsequently challenged by experiments suggesting a role for synaptic NMDARs in excitotoxicity [75,76]. Papouin et al. [13] demonstrated the importance of synaptic NMDARs in synaptic potentiation and excitotoxicity by incubating hippocampal slices with substances that degrade D-serine or glycine. NMDA-induced cell death was significantly reduced by the degradation of extracellular D-serine, a co-agonist of synaptic receptors, whereas degradation of glycine, a co-agonist of extrasynaptic receptors, had little therapeutic effect. This study, without excluding the involvement of extrasynaptic receptors, demonstrates a significant contribution of synaptic receptors to excitotoxicity, as a full inhibition was not reached in both cases [13,23,77]. It seems that the composition of the GluN2 subunit is the determining factor for the induction of neuronal survival or death, but the results of studies vary among authors [78,79,80,81,82]. It remains nevertheless difficult to draw a clear converging conclusion from all these studies. This is especially due to the diversity of protocols set to induce excitotoxic processes. Although harmonization shall be pursued, it appears nonetheless that solely the overactivation of synaptic or extrasynaptic NMDARs is not sufficient to induce the phenomenon of excitotoxicity [77].

## 5. Post-Synaptic NMDA Receptors

The function of glutamatergic transmission is also mediated by post-synaptic NMDARs. In most synapses, they are located close to AMPARs in order to form a functional synaptic unit. Glutamate released from the presynaptic element thus coactivates both types of receptors [9], and the contribution of each to the synaptic currents generated varies from synapse to synapse. The activation of post-synaptic NMDARs is associated with various physiological processes, such as synaptic maturation and plasticity. Therefore, they are present at various densities on synapses. Several studies have shown a differential distribution of NMDAR subtypes at the synaptic membrane: receptors containing the GluN2A subunit are concentrated in the center of the synapse, whereas receptors containing the GluN2B subunit are more perisynaptic [83,84]. This asymmetric distribution has also been investigated functionally in the granular cells of the hippocampal dentate gyrus. Spontaneous, monosynaptic currents pass predominantly through the activation of NMDARs containing the GluN2A subunit and induce little or no activation of peri- and extrasynaptic receptors, in contrast to evoked currents that pass through receptors containing the GluN2B subunit [52,85,86]. However, not all synapses possess the same composition of receptor subtypes. In the hippocampus, for example, the number of GluN2B subunit receptors in pyramidal cell synapses in the CA1 and CA3 regions differs between the left and right hemispheres [83,87]. The degree of NMDAR subtypes variability is related with specific brain regions such as the hippocampus or within a single neuron (e.g., interneuron or pyramidal neuron) and the afferents [87,88,89,90]. Other brain structures, such as the neocortex, also exhibit this afference-dependent asymmetry [91]. The maturation of glutamatergic synapses requires the reorganization of postsynaptic ionotropic receptors. During the first week of postnatal life, synapses contain primarily NMDARs [92]. Several models have been proposed to account for the synaptic remodeling observed during this period. However, everyone unanimously acknowledges that the activity of NMDARs affects the functional maturation of synapses, particularly through the modulation of AMPARs density [93,94,95,96]. These receptors have also been demonstrated to be involved in both (i) the morphological maturation of glutamatergic synapses by regulating the density and morphology of dendritic spines [97,98] and (ii) the organization of neuronal circuits, particularly in the refinement of synaptic connections during maturation [99]. This is indicative of the effect of these receptors on neuroplasticity, the ability of the CNS to reorganize its structure, function, and connections as a response to both intrinsic and extrinsic stimuli. This takes place at multiple levels, including behavioral, cellular, and molecular, and serves as the foundation for learning and memory. Thus, synaptic plasticity reflects the dynamics of connections between neurons throughout an individual’s life. The effectiveness of a synapse can be modulated by two criteria: direction (potentiation or depression) and duration (short- or long-term). NMDARs play an important role in triggering these synaptic plasticity phenomena, in particular long-term potentiation (LTP) and long-term depression (LTD), which have been extensively studied [100,101]. These changes in the strength of synaptic transmission within a neuronal network may form a physiological basis for memory processes. The most well-characterized form of NMDAR-mediated LTP occurs in the hippocampus between CA3 and CA1 pyramidal neurons, a limbic structure that is widely recognized as being involved in learning processes [101,102]. LTP is a process that enhances the effectiveness of the synaptic response induced by the application of a series of high-frequency stimuli or specific substances [19]. This phenomenon cannot be induced solely by glutamate but rather involves the activation of both pre- and postsynaptic neurons, which fulfil the two prerequisite conditions for the activation of NMDARs: the release of glutamate and a robust membrane depolarization, thereby facilitating the expulsion of the Mg^2+^ block. The influx of Ca^2+^ reaches its maximum, triggering a signaling pathway that leads to an increase in the density of AMPARs on the synapse [101,103]. Although this cascade is not completely understood, it involves several proteins, including Ca^2+/^calmodulin-dependent kinase II (CaMKII), which is capable of phosphorylating AMPARs and thereby increasing their conductance [104]. Subsequent to this last stimulation, the evoked potentials can persist at elevated levels for a lifetime of several hours or days. Unlike LTP, LTD can be induced by repeated stimulation of the presynaptic neuron at low frequencies without activation of the postsynaptic neuron. In fact, blocking of NMDARs by Mg^2+^ is incomplete even at resting potentials [105]; a small amount of Ca^2+^ can still enter the cell in response to this low-frequency synaptic stimulation [106,107,108]. The repeated occurrence of this low Ca^2+^ influx triggers the induction of LTD. This results in a decrease in the number of synaptic AMPARs by endocytosis [101,103]. This effect also involves the activation of several proteins, such as protein phosphatase 1 (PP1), whose high affinity for Ca^2+^ promotes its activation at lower concentration [109]. The observed phenomena of exocytosis and endocytosis of AMPARs suggest the existence of a mobile pool of AMPARs that can be “recycled” between the cytoplasm and the cell membrane within a few minutes. These two processes do not occur directly on the synapse but rather at the perisynaptic level, from where the receptors diffuse to reach the postsynaptic density [101]. These synaptic plasticity phenomena may not solely involve structural modifications at the synapse level but also involve the maintenance of these modifications through the process of protein synthesis. Several signaling proteins are activated during LTP/LTD to initiate the transcription and translation of growth factors and proteins required to maintain these modifications. In this manner, LTP and LTD can direct the selective stabilization of certain synapses or suppress others. For instance, the Arc gene is a precursor gene that is capable of orchestrating the translation of mRNA at the dendritic level essential for the polymerization of actin and the stabilization of dendritic spine extension during LTP [110]. Synaptic NMDARs also appear to play a critical role in neuronal survival by activating neuronal survival pathways [111,112]. Studies indicate that blocking NMDARs leads to apoptosis and neuronal degeneration [113,114]. The neuroprotective function that is dependent on NMDAR primarily involves the activation of pro-survival transcription factors and the inhibition of apoptosis [56,115]. Furthermore, activation of synaptic NMDARs promotes the expression of survival genes by activating Ca^2+^-dependent transcription factors such as the CREB protein. Ten genes have been identified as providing neuroprotection by making mitochondria more resistant to oxidative stress. The CREB gene also functions in the synthesis of BDNF, which possesses neuroprotective properties. The activation of synaptic NMDARs also inhibits several mechanisms involved in pro-apoptotic pathways (caspases, PUMA (p53 up-regulated modulator of apoptosis) gene) and suppresses the expression of pro-apoptotic transcription factors such as FOXO (forkhead box protein O) or p53 [116].

## 6. Implications of NMDARs for Neuropsychiatric Disorders

Because of their central role in many physiological processes, NMDARs are naturally involved in several neurological and psychiatric disorders. These pathological processes may be associated with excitotoxicity, altered LTP or LTD, or a receptor hypofunction. In this section, we summarize the various receptor dysfunctions implicated in some of these pathologies.

### 6.1. Stroke

Stroke is the first CNS pathology for which NMDAR dysfunction has been proposed. During cerebral ischemia, the concentration of extracellular glutamate significantly rises to toxic levels. This phenomenon is largely attributed to a reduction or even a reversal in the activity of the transporters that normally remove glutamate from the synaptic cleft [117,118,119] and to an increase in presynaptic vesicular release [120]. This increase in glutamate concentration results in excitotoxic activation of NMDARs, primarily extrasynaptic, particularly those containing the GluN1/GluN2B subunits. The receptor opening induces the activation of pro-apoptotic agents such as calpain, a Ca^2+-^activated protease [121], and inhibits survival factors such as the CREB nuclear transcription factor [66], ultimately leading to cell death. A study conducted in mice also demonstrated that DAPK1 (death-associated protein kinase 1) binds to the C-terminal region of GluN2B subunits, resulting in their phosphorylation and enhancing receptor conductance [122]. However, as discussed above, some studies suggest that synaptic NMDARs may be involved in this excitotoxicity phenomenon. Some NMDAR antagonists that have the capability to inhibit intraneuronal calcium entry have been proposed as therapeutic agents to limit the volume of ischemic lesions. However, most of these molecules, which also affect synaptic transmission, exhibit significant adverse effects, such as hallucinations, respiratory impairment, and memory disorders, among others, thus limiting their utilization [115].

### 6.2. Epilepsy

NMDARs play a major role in the physiopathology of epilepsy and glutamate-mediated neuronal hyperexcitation plays a causal role in the initiation of epileptic seizures [123]. Indeed, the augmentation of extracellular glutamate concentration during seizures has been extensively documented [124]. Among the glutamatergic receptors, NMDARs and AMPARs are important targets for clinical research in this disease. However, their func-tion in ictogenesis remains poorly understood. It has been observed that agonists of NMDARs and AMPARs can induce convulsions [125,126], while antagonists are known to inhibit convulsions in some animal models [127]. Numerous studies have been con-ducted on models of genetic mutations resulting in hyper- or hypofunction of NMDARs. However, the varying clinical phenotypes produced have prevented a definitive categori-zation of these diverse forms of epilepsy [128,129]. Other studies have demonstrated a distinct function for AMPARs through an elevation in their expression, which may be at-tributed to genetic mutations or a process of synaptic plasticity [130,131]. The therapeutic use of molecules targeting glutamatergic receptors has been assayed; however, the major-ity, primarily NMDARs antagonists, have not demonstrated acceptable efficiency and safety [132,133]. Only one AMPARs antagonist, perampanel, has been approved for the treatment of various forms of epilepsy [134].

### 6.3. Anti-NMDAR Encephalitis

Anti-NMDAR autoimmune encephalitis has been identified as an entity that is associated with epilepsy. The diagnosis of anti-NMDAR encephalitis is confirmed by the detection of cerebrospinal fluid antibodies against the GluN1 subunit of the NMDAR. Two potential triggers of autoimmune encephalitides are tumors and viral encephalitis. It was diagnosed in young women with a typical progression of psychiatric symptoms, from subtle behavioral changes such as irritability to full-blown psychosis. These symptoms are followed by motion disorders, autonomic dysfunction, hypoventilation, seizures, and a coma [135]. Anti-NMDAR antibodies are responsible for the internalization of NMDARs, leading to a decrease in their density at the cell surface [136,137]. This decrease is responsible for the inhibition of GABAergic interneurons, which results in the disinhibition of central excitatory pathways and ultimately leads to the frontostriatal syndrome characteristic of this form of encephalopathy [138]. The condition has a higher prevalence among females, with a ratio of approximately 8:2. About 37% of patients are under the age of eighteen at the time of diagnosis. Two possible triggers for the condition are tumors (mainly ovarian teratomas) and herpes encephalitis [139]. The treatments combine immunotherapy with plasmapheresis sessions and immunosuppressive therapy (corticoids, rituximab, and cyclophosphamide) [140].

### 6.4. Alzheimer Disease

As per the World Health Organization, Alzheimer’s disease (AD) is the primary cause of dementia, accounting for 60–70% of cases. The symptoms of this chronic neurodegenerative disease worsen over time, from early forgetfulness to progressive deterioration of language, orientation, and behavior and finally severe memory loss [116]. The underlying cause of the disease is intricate and multifactorial. One or more genetic mutations in the presenilin (PS1, PS2) and amyloid precursor protein (APP) genes cause an early-onset familial form of AD. These mutations affect the synthesis and proteolysis of APP, leading to an excessive production of amyloid β protein (Aβ) [141]. However, the root cause of late-onset sporadic AD remains ambiguous. The primary risk factor is genetic; however, other factors such as ageing, apolipoprotein (Apo) E4 genotype, brain trauma, or certain vascular conditions [142] have also been suggested. The pathophysiology of AD encompasses both structural and functional abnormalities, particularly in the hippocampus and neocortex, which are particularly involved in memory and cognition [143]. As the disease progresses, a multitude of anatomical lesions occur in the brain, including the emergence of senile plaques comprised of amyloid β protein (Aβ) and neurofibrillary tangles containing phosphorylated tau protein, which ultimately lead to neuronal degeneration. These pathological changes are also correlated with the activation of NMDARs and oxidative stress. Alzheimer’s disease is characterized by three main types of histological lesions: neurofibrillary degeneration (NFD), extracellular deposition of amyloid beta protein, and neuronal and synaptic loss [144]. However, cognitive decline begins before this stage, and there is increasing evidence that particular proteins cause early memory impairment by disrupting the LTP and LTD mechanisms [145,146]. Indeed, soluble Aβ oligomers strongly activate metabotropic receptors, leading to AMPAR internalization and LTD. Therefore, physiological NMDA-dependent LTD is inhibited. Moreover, oligomers are responsible for reducing the population of NMDARs, preventing the induction of LTP. In this particular setting, it has been observed that synapses undergo a decrease in thickness, and some may even disappear [101]. Furthermore, the Aβ peptide is thought to play an important role in triggering the excitotoxic process responsible for the neuronal death associated with Alzheimer’s disease via several mechanisms. Initially, the Aβ peptide is responsible for elevating the extracellular concentration of glutamate by enhancing the release of astrocytic glutamate through binding to α7 nicotinic acetylcholine receptors and by reducing its reuptake. The binding to α7 nicotinic receptors may also promote the internalization of synaptic NMDARs by activating protein phosphatase 2B (PP2B), which dephosphorylates and activates striatal-enriched protein phosphatases (STEP). STEP dephosphorylates NMDARs, which leads to the internalization of synaptic NMDARs, further shifting the balance towards extrasynaptic NMDA signaling. Furthermore, an increase in extracellular glutamate and the activation of extrasynaptic NMDARs leads to the production of nitric oxide (NO), which activates a signaling cascade that also leads to mitochondrial fragmentation. This cascade is also implicated in the hyperphosphorylation of the tau protein. Finally, the elevated activity of extrasynaptic NMDARs is also associated with the inhibition of several pro-survival proteins and transcription factors (such as ERK1/2 and CREB) and with the opposite activation of pro-apoptotic factors (such as FoxO3a, p38MAPK).

The activation of extrasynaptic NMDARs is also associated with the increased expression of APP containing the KPI domain, which facilitates its cleavage by β-secretase into Aβ. Aβ peptide is then released into the extracellular space in an activity-dependent manner [115].

### 6.5. Huntington’s Disease

Huntington’s disease (HD) is an autosomal dominant neurodegenerative disorder affecting cognition, motor skills, and mood, caused by a mutation in the gene encoding Huntington’s protein (Htt). This mutant protein (mHtt) causes defects in signaling, synaptic plasticity, and neuron death, especially in the GABAergic spiny neurons of the striatum [147]. Neuronal death appears to be caused by the overactivation of extrasynaptic NMDARs, leading to excitotoxicity [115,148]. Indeed, intrastriatal injections of NMDAR agonists have often been used to reproduce, in animals, the lesions observed in HD [149,150]. Similarly, the overexpression of the GluN2B subunit in striatal neurons in a mouse model of HD leads to their increased degeneration [151]. The mechanisms underlying this excitotoxicity are diverse, with the mHtt protein engaging in a variety of signaling pathways. The outcome is a rise in the levels of extracellular glutamate, and a decrease in the endocytosis of extrasynaptic NMDARs resulting in an increase in their quantity and activity, particularly those containing the GluN3A subunit [115].

### 6.6. Schizophrenia

Schizophrenia is a multifaceted neuropsychiatric disorder that is characterized by a hyperfunction of the dopaminergic system. This ailment leads to a diverse range of cognitive and behavioral manifestations, including “positive” symptoms such as hallucinations and delusions, “negative” symptoms such as apathy and anhedonia, and “cognitive” symptoms such as attention and memory deficits [152]. Over the course of several decades, extensive research has identified excessive striatal presynaptic dopamine release as a prevalent final pathway for the elicitation of positive symptoms [153]. However, the underlying pathophysiology of negative and cognitive symptoms, which often precede positive ones, remains unresolved. The first clue of the involvement of NMDARs in schizophrenia came from the observation that some molecules, such as phencyclidine (PCP) and ketamine, are able to antagonize NMDARs by blocking the ion channel, to induce psychotic and negative symptoms. Furthermore, cognitive impairments similar to those described in schizophrenia may be observed in healthy individuals [154,155]. These symptoms are intensified in schizophrenic patients [156,157]. The clinical expression of anti-NMDAR encephalitis includes marked psychiatric symptoms and cognitive impairment. This also suggests the involvement of NMDARs in schizophrenia [139]. However, these patients also exhibit movement disorders, convulsions, and autonomic instability. The hypothesis put forward to explain this phenomenon is that these drugs block synaptic NMDARs in a population of GABAergic interneurons (disinhibition theory). This results in a reduction in their activity and consequently in the release of GABA, thereby resulting in the disinhibition of glutamatergic neurotransmission in the prefrontal cortex. The release of glutamate would stimulate the AMPARs in the pyramidal cells, leading to the state of hyperactivity described. The dopaminergic hyperactivity observed in schizophrenia could be attributed to a hypofunction of NMDARs. Several studies have identified this hypofunction in fast-spiking corticolimbic (FS) neurons. It is believed to result from several major genetic and/or environmental factors that lead to schizophrenia. Certain genetic mutations, such as the overexpression of neuregulin (NRG) or the inactivation of the gene encoding the α7 nicotinic acetylcholine receptor (α7AChR), which causes a reduction in the concentration of D-serine in the synaptic cleft, are likely to cause NMDAR hypofunction in GABAergic neurons. Likewise, some environmental factors, such as anti-NMDA antibodies (which can be synthesized following infections such as toxoplasmosis or herpes), inflammation, and oxidative stress, can also negatively regulate their activity. However, all these heterogeneous and multifactorial mechanisms are still not fully understood [152,158].

### 6.7. Depression

Depression is a major cause of disability worldwide. It affects approximately 280 million people worldwide as per the World Health Organization. Approximately 50% of patients with major depressive disorder respond to standard treatments that target the monoaminergic system. However, the pathophysiology of the disorder is not completely understood. The conventional theory of monoamine depletion (serotonin, norepinephrine, and/or dopamine) does not explain the unsatisfactory therapeutic response of several patients to antidepressants. In recent years, the role of glutamate neurotransmission and NMDARs in depression has attracted the attention of the scientific community. Numerous factors suggest a link between depression and NMDAR dysfunction. In some depressive patients, abnormalities in the expression of genes encoding the receptor have been detected [159,160]. Various stress factors induce excessive NMDAR activity [161]; finally, some receptor channel-blocking antagonists, such as ketamine, have antidepressant effects [162,163]. In 2019, an intranasal S-ketamine-based antidepressant (Spravato^®^) was approved in the European Union for use in combination with other antidepressants for the acute short-term treatment of depressive symptoms. Ketamine is believed to block NMDARs, containing the GluN2B subunit, located in parvalbumin-expressing GABAergic interneurons. This would increase the levels of extracellular glutamate and increase the number of synapses in the prefrontal cortex. The activation of AMPARs by glutamate in this area via signaling pathways involving, in particular, the mTOR protein, would induce the synthesis of new proteins such as BDNF and the GluA1 subunit. These proteins are believed to be responsible for the rapid antidepressant effects of ketamine [158,164,165].

### 6.8. Neuropathic Pain

Neuropathic pain is chronic and a persistent condition that arises from damage of the peripheral and/or central nerves. It is now widely acknowledged that NMDARs play a significant role in the initiation and maintenance of chronic pain states [166]. NMDARs are also expressed presynaptically, particularly in the central terminals of primary sensory neurons in the dorsal horn of the spinal cord. Nonetheless, these neurons are typically in a state of quiescence and are not actively engaged in the transmission of physiological nociceptive signals. Recent studies indicate that the activity of presynaptic NMDARs in the spinal cord is enhanced in neuropathic pain resulting from traumatic nerve injury, chemotherapy, or calcineurin inhibitors [167,168]. The heightened activity of receptors has the potential to enhance glutamate release from primary afferent terminals on neurons located in the dorsal horn of the spinal cord. It is associated with the binding of the receptor to the C-terminus of α2δ-1, an auxiliary subunit of Ca^2+^ channels [169]. This binding is accountable for augmenting presynaptic NMDA activity by facilitating synaptic trafficking of α2δ-1 NMDAR complexes and/or reducing receptor blockade by Mg^2+^ ions. Moreover, the binding of gabapentinoids such as gabapentin and pregabalin to α2δ-1, can normalize pre- and postsynaptic receptor activity, thereby reducing neuropathic pain [167,170].

### 6.9. Opioid-Induced Tolerance and Hyperalgesia

Opioids constitute the most potent class of analgesics utilized to alleviate acute and chronic pain. These molecules include alkaloids extracted from poppy seeds (morphine, codeine), their semi-synthetic derivatives (oxycodone, hydromorphone, oxymorphone, and synthetic phenylpiperidines (meperidine, fentanyl)), and synthetic pseudo-piperidines such as methadone [171]. Their action is mediated by the activation of three major classes of opioid receptors (µ, δ, and κ), which are widely distributed in the spinal cord (dorsal horn) as well as in specific cerebral structures, specifically the periaqueductal gray matter and the locus coeruleus. The activation of these GPCRs induces analgesia via various intracellular pathways, leading to an inhibited release of glutamate and neuropeptides such as substance P in primary afferent fibers as well as hyperpolarization of ascending projection neurons. These molecules also have a central analgesic effect through activation of the descending analgesic pathway [172]. Nonetheless, prolonged use of these molecules may also result in tolerance and hyperalgesia (exacerbation of nociceptive transmission), whose mechanisms remain ambiguous but that surely involve NMDARs. Indeed, NMDARs are located presynaptically at the central termini of primary afferent fibers and postsynaptically on neurons located in the dorsal horn of the spinal cord [173]. Several studies in rodents have shown that intrathecal or intravenous administration of the NMDAR antagonist MK-801 reduces or prevents the onset of opioid-induced hyperalgesia (OIH) [174,175]. It has been demonstrated in a mouse model of OIH that chronic morphine administration leads to an elevation in the expression of the GluN1 subunit and that this effect can be mitigated by blocking NMDARs [176]. Moreover, it has been demonstrated that chronic administration of morphine and NMDARs antagonists in mice decreases the transcription of β-arrestin2 (Arrb2), a protein that is overexpressed in the periaqueductal gray matter, cortex, and striatum during analgesic tolerance and is implicated in the desensitization of opioid receptors [176,177]. These results demonstrate that Arrb2 activity regulated by NMDARs is involved in both OIH and tolerance, strengthening the link between these two phenomena [178]. Electrophysiological experiments have demonstrated that chronic opioid administration increases the activity of presynaptic NMDARs in the dorsal horn of the spinal cord and decreases the activity of postsynaptic NMDARs [179]. The authors hypothesize that chronic opioid administration can induce translocation of a protein kinase C (PKC) to the plasma membrane, which is responsible for activating presynaptic NMDARs by removing Mg^2+^ that blocks the ion channel. This induces the receptor to move towards the plasma membrane, leading to an enhanced release of glutamate. Combined with the inhibition of the glutamate reuptake system, these mechanisms lead to an increase in glutamate concentration in the synaptic cleft, resulting in synaptic overstimulation that could lead to hyperalgesia [178]. These mechanisms and the many other molecular pathways involved in these phenomena are the subject of growing literature, making NMDARs one of the molecular targets for the prevention of hyperalgesia.

## 7. NMDARs Pharmacology

Since hypo- and hyperactivation of NMDARs is associated with the development of several neurological and psychiatric pathologies [180], these receptors represent a potential therapeutic target of high value. This implication has resulted in the development of allosteric modulators and molecules capable of blocking the ion channel. NMDAR is a complex pharmacological target with at least four different binding sites: an intracanal site, also known as the “PCP site,” which blocks the efflux of calcium, a glutamate-binding site on the GluN2 subunit, a co-agonist (glycine or D-serine) binding site on the GluN1 subunit, and several allosteric modulation sites. D-cycloserine, in addition to other NMDAR glycine agonists, such as glycine, serine, and D-serine, has been examined in the treatment of neurodevelopmental disorders, in particular schizophrenia. Preliminary studies suggest that further research into D-cycloserine as a possible treatment for certain neurodevelopmental diseases is needed to identify if glycine site agonists of the NMDAR are a viable treatment paradigm [181]. In this paragraph, we have decided to focus our analysis on ion channel blockers because glutamate agonists, antagonists, and allosteric modulators lack specificity and have not demonstrated clinical success over time [181]. Indeed, channel blockers have the advantage of targeting only NMDARs whose channel is open, allowing them to be visualized, as in the case of radiotracers. They also block them for therapeutic purposes. Because of this, the structure of channel blockers has served as a basis for the synthesis of drugs used for diagnostic or therapeutic purposes. Several molecules may inhibit NMDAR by blocking the ion channel. These channel blockers can be divided into three categories, depending on how they interact with the ion channel:

- Sequential blockers or foot-in-the-door, which can only bind to the open channel and, once bound, prevent it from closing, include aminoacridine and tetrapentylammonium derivatives [182,183].
- Partial blockers, which obstruct the channel without completely preventing its opening, include amantadine and memantine [184].
- Trap blockers or total blockers that remain trapped in the pore and keep it closed include ketamine, phencyclidine (PCP), and dizocilpine (MK-801) [185].

**Figure 3:**
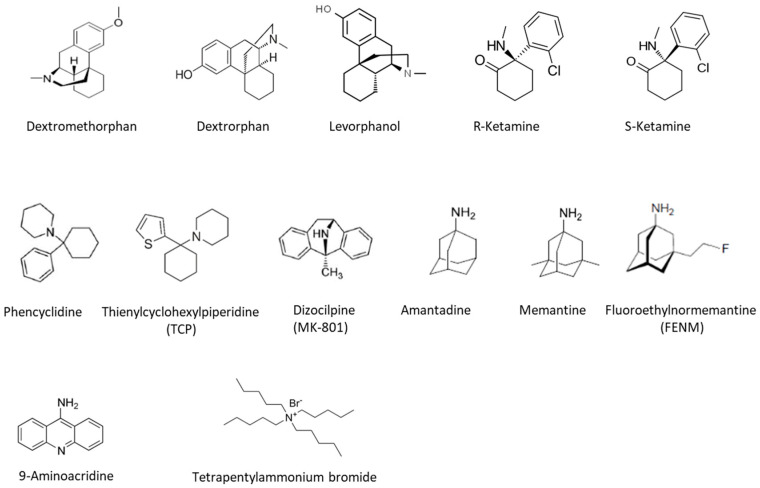
Chemical structures of various NMDA receptor channels blockers.

The binding site of these molecules (Figure 3) to the “PCP site” is intimately associated with the channel domain of the NMDA receptor. It partially overlaps with the Mg^2+^ binding site between the ABD domain and the C-terminal domain. In order for the ligands to bind, the Mg^2+^ must be absent from the channel. The antagonists adopt an “inverted triangle” configuration, and their binding occurs via hydrophobic interactions with the M3 transmembrane helix and a polar bottom mainly contributed by the QRN (glutamate, arginine, asparagine) site at the tip of the pore loop [186]. It is worth noting that these molecules are only able to access the channel when it is open [184]. Since the ion channel is a highly conserved region between receptor subtypes, blockers of the channel are thought to be non-selective [8]. However, certain antagonists, such as memantine, preferentially target specific receptor subpopulations [187]. These molecules show neuroprotective effects in some animal models of neurological diseases linked to excitotoxicity. Compounds with high and medium affinity for the receptor (ketamine, MK-801, PCP, TCP) are dissociative anesthetics, meaning that they make people feel separate from reality and whose clinical use is limited by their psychomimetic side effects [8].

### 7.1. Dextromethorphan

Dextromethorphan is a dextrorotatory isomer of levorphanol and a codeine analogue that has no analgesic properties at therapeutic doses but has been used as a cough suppressant for over 40 years. It was the first NMDAR channel blocker to be approved by the FDA (Food and Drug Administration) in 1958. Its bioavailability is 10% due to rapid hepatic metabolism to the opioid agonist dextrorphan, the main active metabolite. Dextrorphan undergoes rapid glucuronidation, resulting in an ionic metabolite that passes through the blood–brain barrier. Besides its NMDAR antagonist activity, its pharmacological action is due to an agonist effect on sigma-1 receptors and an inhibitory effect on serotonin and norepinephrine reuptake [188]. In 2022, the FDA approved the combination dextromethorphan/bupropion to serve as a rapid-acting antidepressant in patients with major depressive disorder.

### 7.2. Ketamine and Esketamine

Ketamine is a drug mainly used as an anesthetic. It is a fast-acting general anesthetic, administered intravenously or intramuscularly, that acts essentially by blocking NMDARs at concentrations between 2 and 50 μM [189]. It produces an anesthetic state different from that observed with drugs that act predominantly at the GABA receptors (barbiturates, propofol, benzodiazepines, etc.). This type of anesthesia is known as “dissociative anesthesia” and involves a reduction in activity in the neocortex and subcortical structures (thalamus) and an increase in activity in the limbic system and reticular substance. This causes a cataleptic state in which the patient’s eyes remain open, corneal and photomotor reflexes are maintained, and a characteristic nystagmus is produced. This compound is particularly effective for short-term anesthesia. It is also possible to achieve anesthesia lasting several hours with repeated injections or intravenous infusions. It is also used as an anesthesia inductor before the administration of other anesthetic agents or as a potentiator of low-potency anesthetic agents such as nitrous oxide [21]. Ketamine also has an antidepressant effect, at lower doses, leading to the launch in 2020 of Spravato^®^, a nasal spray solution containing the S (+) enantiomer of ketamine, or esketamine, for the treatment of resistant depression after the failure of other classes of antidepressants. The S (+) enantiomer exhibits 3–4-fold higher affinity for the PCP site compared to the R enantiomer [186].

### 7.3. PCP, TCP, and MK-801

Phencyclidine (PCP, also known as angel dust by drug addicts), thienylcyclohexylpiperidine (TCP), and dizocilpine (MK-801) are high- and medium-affinity antagonists of the intracanal site of NMDARs. These molecules have demonstrated neuroprotective properties in preclinical models of excitotoxicity [190]. The aforementioned substances possess a dissociative anesthetic effect. However, unlike ketamine, their higher affinity and prolonged half-life restrict their use as such in humans due to their potent psychomimetic properties [8,188,191]. However, they are considered reference molecules for studying the in vitro affinity of many compounds for the PCP site [192,193,194,195].

### 7.4. Memantine

Memantine is an amantadine derivative that possesses neuroprotective properties that have been approved by the FDA and the European Union for the treatment of AD. Despite having interesting properties in mice, it has been considered that the medical benefit provided by memantine is low. Memantine exhibits a lower affinity for the PCP site compared to dissociative anesthetics such as dizocilpine. Moreover, despite having a similar affinity to ketamine, memantine exhibits distinct clinical effects. Several studies have been conducted in order to explain this difference. It may be due to interaction with other pharmacological sites, especially at high doses. Memantine can bind to acetylcholine, serotonin 5-HT3, sigma-1 receptors, or voltage-gated sodium channels, whereas ketamine can bind to dopaminergic D2, serotonergic 5-HT2 receptors, and HCN1 channels. However, activity of metabolites inhibiting particular populations of NMDA receptors cannot be excluded. The interaction of memantine with NMDARs is predominantly observed in open channels in the presence of a prolonged rise in glutamate concentration typically lasting several minutes, as observed in extrasynaptic receptors. Numerous studies have demonstrated that memantine exhibits a preferential inhibition of extrasynaptic receptors [196,197], which may account for the superior tolerability of this molecule in comparison to other antagonists such as ketamine, which exhibit a preferential inhibition of synaptic receptors. Nevertheless, the rationale behind this apparent preference for extrasynaptic receptors remains unclear [184].

### 7.5. Fluoroethylnormémantine

Fluoroethylnormemantine (FENM) is a fluorinated analogue of memantine. This molecule was ^18^F- radiolabeled to study its biodistribution in vivo [198,199,200]. More recently, preclinical studies have evaluated FENM in neuroprotective [201], antidepressant effects and its effectiveness in various behavioral disorders [202,203]. FENM also displays synergistic protection in combination with a sigma-1 receptor agonist in a mouse model of Alzheimer’s disease [204]. The combination of autoradiography and ex vivo immunohistochemistry demonstrated colocalization between radiolabeled FENM and NMDARs in the cortex and cerebellum of rats. Furthermore, the injection of (R, S)-ketamine prior to autoradiography prevented FENM binding. Thus, FENM binds to the PCP site in the ion channel and has a lower affinity for this site compared to ketamine [198,199]. In vivo studies performed in 2021 concluded that FENM enhanced cognitive function and displayed neuroprotective properties in a mouse model of AD. In this study, Couly and colleagues [201] found that the administration of FENM prevented the onset of memory impairment and oxidative stress by reducing lipid peroxidation and cytochrome c release. Moreover, a decrease in inflammation processes in the hippocampus and cortex was demonstrated. Unlike memantine, FENM improved spatiotemporal orientation. A recent study has demonstrated that drug combinations based on FENM and Sigama-1 receptor agonists may lead to highly effective and synergistic protection in AD, particularly in short-term memory [204]. Furthermore, two other preclinical studies have shown the ability of FENM to reduce stress-related behavioral dysfunction and to modify AMPAR-mediated hippocampal activity in the same way as ketamine, suggesting a common neurobiological mechanism. However, this remains unclear [33,34].

## 8. Conclusions

Since the NMDAR system coexists with other glutamate systems, it represents a highly complex neurotransmitter system. Furthermore, the distribution of NMDAR at different levels of the CNS (pre-, post-, and extra-synaptic and various brain regions) makes it difficult to determine the exact role of NMDA receptors at both physiological and pathophysiological levels. Furthermore, the biochemical organization of the receptors into four subunits increases the complexity of the system. Nevertheless, this encompassing complexity has not hindered the development of pharmaceuticals and imaging probes that exert their effects on these receptors and particularly at the level of the ion channel. With regard to the future marketing of new molecules, the challenge will be to find new chemical entities capable of blocking the ion channel without causing psychotomimetic side effects. In this context, efforts should be made to find new molecules capable of interacting at various structural levels. These interactions can be validated both by pharmacology and molecular modeling experiments but also in animal models.

## Data Availability

Data sharing is not applicable.
